# Analysis of the pattern of maxillofacial fractures in north western of Iran: A retrospective study

**DOI:** 10.4103/0974-2700.76837

**Published:** 2011

**Authors:** Ali Hossein Mesgarzadeh, Mohamadreza Shahamfar, Samira feizi Azar, Jafar Shahamfar

**Affiliations:** Department of Oral and Maxillofacial Fractures, Tabriz School of Dentistry, Iran; 1 Department of Orthodontics, School of Dentistry, Iran; 2 Tabriz University of Medical Sciences, Iran; 3 Department of Community Medicine, Tabriz School of medicine, Tabriz, Iran

**Keywords:** Maxillofacial fracture, retrospective study, road traffic accidents

## Abstract

**Background:**

Maxillofacial fractures can lead to substantial long-term functional, esthetic and psychological complications.

**Aim:**

The aim of this study is to evaluate these injuries in a Turkish Iranian population.

**Materials and Methods:**

A retrospective study of 170 patients with 210 maxillofacial fractures admitted to the emergency department of a central referral emergency hospital in the area over a 5 year period is presented. Patients’ data included demographic information, etiology, site and associated injuries and complications.

**Results:**

Road traffic accident was the commonest cause (40%) and the age group of 21-30 comprised the biggest group (30%). Mandibular fractures outnumbered midface fractures (150 vs. 60). Ramus (21.5%) and zygoma (26.5%) were the commonest fracture regions respectively in mandible and midface. Male: female ratio was 3.8:1 Almost half of patients (46%) had sustained associated injuries most of which was soft tissue laceration of the face (17.5%). 22 patient (13%) had associated complication and the hemorrhage was the commonest form of that (9%).

**Conclusion:**

It seems that road traffic accidents continue to be the leading cause of maxillofacial fractures and there is an urgent need to implement enhanced regulations and monitoring on motor vehicular traffic.

## INTRODUCTION

Maxillofacial injuries can pose considerable long-term functional, esthetic, and psychological complication.[1,2] These injuries may also pose a substantial economic consequence for the patients as the treatment may require a complex procedure.

It has been said that maxillofacial fractures very markedly from one country to another and even within the same country. This considerable variability is due to a range of factors such as the prevailing socioeconomic, cultural and environmental as well as age and sex distribution of the population. Worldly speaking the main causes of maxillofacial fractures are road traffic accidents (RTAs), assaults, falls, sports-related injuries and wars.[3] A WHO statistics report indicated that each year one million people die and between 15 and 20 million are injured due to RTAs.[4]

In Iran death from RTA is the leading cause of mortality up to the age of 29.[5] While some features in the pattern of maxillofacial fractures are widely established, e.g., male predominancy,[6–8] other aspects have varied. International studies from Jordan,[9] Singapore,[10] and New Zealand[11] have reported RTAs as the most common cause of maxillofacial fractures, while in the USA,[12] Sweden,[13] and Finland[14] assault has been reported as the leading etiological factor. A clearer picture of the etiologic and demographic patterns of maxillofacial injuries can assist health care professionals to deliver optimal management and treatment planning for the plethora of patients affected by traumatic maxillofacial injuries. These epidemiological data can furthermore be utilized to help develop appropriate preventive measurements.

Despite the increasing frequency of morbidity and mortality associated with maxillofacial fractures in Iran, little has been published in this regard. The previously published studies[15–17] have addressed this issue on different parts of Iran. However, we know of previous literature that trends in maxillofacial trauma are largely modulated by time and location. This is especially important since Iran represents a vast country with different ethnic, cultural, and environmental backgrounds. The aim of the current retrospective study was to investigate the pattern of maxillofacial fractures in northwestern Iran (west Azerbaijan) over a 5-year period (2001–2006) with special consideration for age, gender type, site, etiology and associated injuries, and complications.

## MATERIALS AND METHODS

West Azerbaijan is located in the north western Iran in a mountainous and cold climate zone. It has a total area of 37,411 km^2^ and according to the official census data the total population is estimated to be 2.873 million distributed in a mosaic pattern (*Source*: Iranian Statistical Center). Our hospital is a central referral emergency hospital in the area and all kinds of emergency patients are referred to this hospital. Prior to the research, protocol of the study was approved by the corresponding university review board. The records and radiographs of patients who were referred and hospitalized for treatment of maxillofacial fractures over a 5-year period (2001–2006) were reviewed. Patients who had either died before treatment or had been referred to other facilities were excluded. Data collected from hospital records included name, gender, date, site, age, cause, type of fracture and associated injuries, and complications.

The fractures of the mandible were grouped as condylar, coronoid, angle, body, ramus, symphis, parasymphis, and dentoalveolar fractures. The fractures of the middle face included Le fort I, II, III, zygoma, zygomatic arch, nasal complex, orbital wall, orbital blow out, and dentoalveolar fractures. The concomitant injuries to body were registered according to the site. The etiological factors were classified into seven categories, namely RTA, fall, assault, sport, industrial, animal impact, and shotgun. The associated complications were also noted as pulmonary, cardiovascular neurological, and hemorrhage. Twenty records of patients were excluded because of insufficient data, and assessment was performed on 170 patients.

## RESULTS

### Demographic distribution

**Figure 1:**
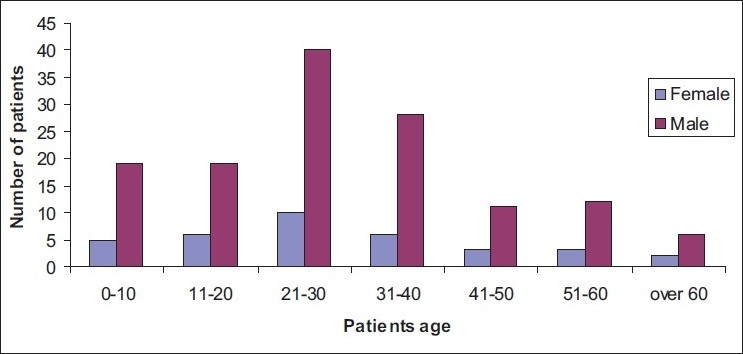
Patients’ age and sex distribution

During a 5-year period, a total of 170 patients with 210 fractures were studied. Each patient had a mean of 1.2 fractures. The age and sex distribution of patients are described in Figure 1. Patients’ age ranged from 5 to 68 with a mean of 29 ± 15. Male patients ranged from 6 to 68 (mean= 29) and female patients from 5 to 65 (mean = 29). The age group 21–30 constituted the biggest group of patients representing 30% of total population. There were an overwhelming male predominantly in all age groups with an over all ratio of 3.8:1.

### Etiology

**Figure 2:**
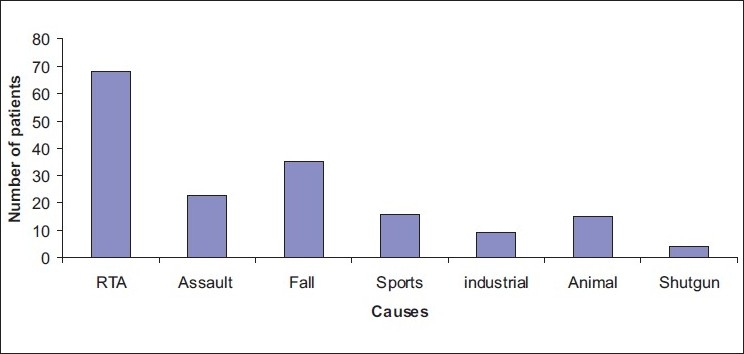
Etiology of maxillofacial fractures in 170 patients

As detailed in Figure 2 the RTAs were the most frequent cause (68; 40%) of maxillofacial fractures. The remaining causes included falls, assaults, sports, industrial, animal, and shotgun accidents in, respectively, descending order. Table 1 describes an etiological approach to the site of the fractures in patients. RTAs and shotgun accidents constituted the most and least frequent causes of fractures in patients both for the mandible and the midface.

**Table 1:** Distribution of patients according to site and cause of fracture

Site Etiology	Manbile no of patients (%)	Midface no. of patients (%)	Mandible + Midface No. of patients (%)	Total (%)
RTA	49 (29)	15 (9)	4 (2.5)	68 (40)
Falls	32 (19)	2 (1)	1 (0.5)	35 (20.5)
Assaul	16 (9.5)	5 (3)	2 (1)	23 (13.5)
Sports	12 (7)	3 (1.5)	1 (0.5)	16 (9.5)
Animal	11 (6.5)	4 (2.5)	–	15 (9)
Industrial	7 (4.5)	2 (1)	–	9 (5)
Shotgun	3 (1.5)	1 (0.5)	–	4 (2.5)
Total	130 (77)	32 (18.5)	8 (4.5)	170 (100)

### Fracture sites

There were 150 (71.5%) mandibular fractures and 60 (28.5%) midface fractures. The differential sidewise distribution of fractures revealed that ramus was the most frequent region (21.5%) in fractures involving mandible [Table 2]. This was followed by the fractures of the condylar region (19.5%). Coronoid on the other hand was the least common area in the mandible to be affected by fracture [Table 2]. Furthermore, analysis of the midface fractures indicated that zygoma fractures constituted the biggest group (26.5%) while orbit fractures (orbit’s floor and walls) were in the second stand [Table 3].

**Table 2:** Locations of mandibular fractures

Location	No. of patients (%)
Condyle	29 (19.5)
Ramus	32 (21.5)
Body	15 (10)
Angle	23 (15)
Parasymphis	16 (10.5)
Symphis	14 (9.5)
Coronoid	6 (4)
Dentoalveolar	15 (10)
Total	150 (100)

**Table 3:** Locations of midface fractures

Location	No. of patients (%)
Zygoma	16(26.5)
Zygomatic arch	8 (13.5)
Nasal complex	5 (8.5)
Orbital wall	10 (16.5)
Blow out	4 (6.5)
Dentoalveolar	3 (5)
Le Fort I	4 (6.5)
Le Fort II	8 (13.5)
Le Fort III	2 (3.5)
Total	60 (100)

Le fort III was the least common (3.5%) fracture of the midface.

### Associated injuries and complications

Almost half of patients (78, 46%) had suffered concomitant injuries to other parts of body. Soft tissue laceration (17.5%) was the commonest form of associated injuries. Pelvic fracture was the second frequent injury (9%); while abdominal injury accounted for the least frequent one (2%, Table 4).

**Table 4:** Patients with associated injuries

Injury	No. of patietnts(%)
Soft tissue laceration of the face	30 (17.5)
Abdominal injury	3 (2)
Chest injury	9 (5)
Cervical spine injury	4 (2.5)
Pelvic injury	15 (9)
Ear injury	11 (6.5)
Eye injury	6 (3.5)

Twenty two patients (13%) were affected by associated complications. Hemorrhage (9%) was the commonest complication associated with maxillofacial fractures while cardiovascular event (0.6%) was the least frequent one [Table 5].

**Table 5:** Patients with associated complications

Complication	No. of patients (%)
Hemorrhage	15 (9)
Respiratory problem	3 (1.7)
Cardiovascular problem	1 (0.6)
Neurological problem	3 (1.7)

## DISCUSSION

The characteristics of maxillofacial fractures depend very much on various factors such as geographical location, culture, and socioeconomic background of the communities. However, epidemiological surveys across the world have revealed that some aspects of the facial fracture patterns remain similar among the various nations. The male predominance as also observed in our study is almost a universal finding reported from other countries such as Canada,[18] Poland,[8] Nigeria[7] and the UAE.[19] We found a male:female ratio of 3.8:1. This ratio is comparable with studies from England,[20] France,[20] India,[21] and Nigeria.[22] However, two previous studies from different regions of Iran have found a ratio of 8:1[17] and 12:1.[16] This surprisingly high range of difference may be attributed to the fact that in mountainous north western Iran due to the environmental and cultural backgrounds women are much more involved in outdoor activities (driving, etc.) resulting in their increased vulnerability to fracture accidents.

On the other hand, it is interesting to note that our male: female ratio is in close harmony with the reported ratio (3:1) in the neighboring country, Turkey. This harmony is specially conceivable when we take into account the fact that the population in our study was mainly predominated by a Turkish–Iranian population who reside in the northwestern of Iran, indicating the influence of ethnicity and culture on the pattern of maxillofacial fractures. If we consider male-to-female proportion as an indirect index for social and economic activities, we may conclude up trending of Iranian and Turkish women in outdoor socioeconomic activities, though this may not be the only reason.

In the current study as also found in other countries,[19,21,22,23,24,25,26] the peak incidence of fracture was in the age range of 21–30. It has been shown that in general young people suffer more from trauma than elder people.[8,9,23,24] This is conceivable because the third decade of life represents an active period when individuals are more energetic involved in high speed transportation and outdoor activities which account for a major proportion of maxillofacial traumas.

In coincidence with the changes in the community lifestyle, industrialization, transportation, and legislative measures, the causes of maxillofacial fractures also tend to change. As a result etiologies differ in various parts of the world. In most developed countries of Europe and North America,[12–14] violence and sports are increasingly replacing traffic accidents while in many developing areas traffic accidents remain the dominant cause.[7,21] In Iran, RTAs are considered to be the second highest cause of mortality (the highest is coronary heart disease) and the data for period 1995–2000 indicate that there has been an estimated 8% annual increase in mortality due to traffic accidents. Traffic accidents have also increased by almost 15% from 2001 to 2003.[27] In the current study, RTAs were the commonest cause and made up 40% of all the incidences. Although when compared with the reports from highly developed countries,[12] this figure is relatively high, this is considerably lower compared with the previous reports from Iran,[15–17] other developing countries[19,21,23] and also from the neighboring country, Turkey. This difference between the two neighboring population and the observed decrease in the rate of RTA in Iran may furthermore be attributed to the influence of geo-demographic and cultural factors. On the other hand, the reason may be attributed (though not limited) to the fact that from 2003, the Iranian police department initiated a program of increased surveillance on compliance with seat bell fastening, helmet wearing, speed control, and road safety measurements to combat the situation. The second and third most common etiologies of fractures in the current study were falls and assault which compared favorably with other reports from the regional countries such as India,[21] Pakistan,[28] and the UAE.[19] However, it should be noted that some victims of assault may state fall instead of violence as the cause of fracture and thus contribute to this sequence.

Alcohol abuse has been reported to be linked to facial injuries from violence in United Kingdom,[29] Sweden,[30] and Finland.[31] It is noteworthy to mention that in our study we found no reported case of alcohol-related violence and this may be related to the strict rules regarding alcohol consumption an Islamic countries as also reported in UAE.[19]

It has been said that in the maxillofacial region, the mandible is more vulnerable than the zygomaticomaxillary complex perhaps because of its position in the face and its prominence. The osteology of mandible, various muscle attachments and their influence, and the presence of developing or completed dentition all play a role in the mandible’s weaknesses.[32] In the current survey, the mandibular fracture (71.5%) outnumbered those of the midface (28.5%) by 2.5 times. This correlates positively with the other previous reports.[15,19,22] While the higher frequency of zygoma fracture in the midface is conceivable because of its prominence and vulnerability during traffic accidents, it is interesting to note that in the mandible condylar and ramus fractures were the commonest sites which may be a reflection of the background etiology of traffic accidents.

In our study, hemorrhage was the commonest form of the associated complications. A variety of incidence rates have been reported in the literature regarding the life-threatening bleeding following maxillofacial trauma.[33,34] Although our aim was not to specifically investigate the hemorrhage incidents, our finding of 9% is relatively higher than previous reports.[4,33] However, it must be emphasized that these studies have mainly registered very massive (fatal) bleedings while in our hospital’s data moderate bleedings might also have been incorporated. Soft tissue laceration of the face, on the other hand, accounted for the biggest group of associated injuries which compare favorably with a previous report from an urban area of Nigeria.[22]

Finally, it has to be noted that the data from the current study was collected from the emergency department while some patients might have been directly admitted to maxillofacial department and therefore be omitted from our records.

## CONCLUSION

In conclusion, it seems that RTAs remain the biggest etiological factor of maxillofacial fractures in Iran. The demographic pattern is in general similar to those of the literature. This includes the higher incidence of fractures in men than women and also in the age span of 20–30. There seems to be an urgent need for enhanced monitoring and regulation on motor vehicles to reduce the morbidity and mortality associated with RTAs. It is hoped that epidemiological surveys, such as the one presented here will help the health care professions and policy makers in planning future programs of prevention and treatment.
